# P09 Genomic characterization of nasal *Enterococcus faecalis* carrying linezolid resistance in healthy hosts identifies zoonotic transmission

**DOI:** 10.1093/jacamr/dlae136.013

**Published:** 2024-08-23

**Authors:** Idris Nasir Abdullahi, Carmen Lozano, Myriam Zarazaga, Javier Latorre-Fernández, Søren Hallstrøm, Astrid Rasmussen, Marc Stegger, Carmen Torres

**Affiliations:** Area of Biochemistry and Molecular Biology, OneHealth-UR Research Group, University of La Rioja, Logroño, Spain; Department of Medical Laboratory Science, Faculty of Allied Health Sciences, Ahmadu Bello University, PMB 05 Zaria, Nigeria; Area of Biochemistry and Molecular Biology, OneHealth-UR Research Group, University of La Rioja, Logroño, Spain; Area of Biochemistry and Molecular Biology, OneHealth-UR Research Group, University of La Rioja, Logroño, Spain; Area of Biochemistry and Molecular Biology, OneHealth-UR Research Group, University of La Rioja, Logroño, Spain; Department of Bacteria, Parasites and Fungi, Statens Serum Institut, Copenhagen, Denmark; Department of Bacteria, Parasites and Fungi, Statens Serum Institut, Copenhagen, Denmark; Department of Bacteria, Parasites and Fungi, Statens Serum Institut, Copenhagen, Denmark; Antimicrobial Resistance and Infectious Diseases Laboratory, Harry Butler Institute, Murdoch University, Perth, Australia; Area of Biochemistry and Molecular Biology, OneHealth-UR Research Group, University of La Rioja, Logroño, Spain

## Abstract

**Background:**

Linezolid resistance in *Enterococcus faecalis* (a normal gut bacterium) is increasingly considered a critical public health concern, hence the need to understand their genomic contents and dissemination patterns.

**Methods:**

Illumina-based sequencing was used to characterize seven linezolid-resistant (LZD^R^) *E. faecalis* strains obtained from the nares of healthy humans and animals in Spain. Also, the relatedness of the strains with publicly available genomes of *E. faecalis* with similar lineages and AMR determinants was accessed by core-genome SNP analysis.

**Results:**

*E. faecalis* was identified in 32.5% of 40 pigs, 30% of 10 pig farmers, 5.9% of 34 dogs and 4.9% of the 41 dog owners. Of the *E. faecalis* strains, only five, one and one strains from pigs, a pig farmer and a dog, respectively, were *optrA*-positive. All the *optrA* were chromosomally located and variants (types 5, 7 and 15) except one that was a WT strain (from a pig). All the strains carried between 16 and 35 virulence genes associated with ≥1 intact prophage. All the enterococci carried ≥1 plasmid replicon (1–5) carrying tetracycline and chloramphenicol resistance genes. SNP-based analyses identified closely related *E. faecalis* strains from a pig and pig farmer on the same farm (SNP=4). Moreover, *E. faecalis* ST32, ST59 and ST474 strains from pigs were related to those previously described from humans (sick and healthy) and cattle in Spain, Belgium, and Switzerland (SNP range 43–86).

**Conclusions:**

These findings strongly suggest the zoonotic transmission of LZD^R^*E. faecalis* and potential international dissemination. These highlight the need to strengthen molecular surveillance of linezolid-resistant enterococci in all ecological niches and body parts to direct appropriate control strategies.

